# From promise to practice: a guide to developing pooled procurement mechanisms for medicines and vaccines

**DOI:** 10.1186/s40545-023-00574-9

**Published:** 2023-06-14

**Authors:** Koray Parmaksiz, Roland Bal, Hester van de Bovenkamp, Maarten Olivier Kok

**Affiliations:** 1 Erasmus School of Health Policy & Management, Erasmus University Rotterdam, Oudlaan 50, 3062 PA Rotterdam, The Netherlands; 2 Health Sciences, VU University Amsterdam, Amsterdam, The Netherlands

**Keywords:** Pooled procurement, Joint procurement, Bulk purchasing, Group procurement, Centralized procurement, Developmental stages, Organizational life cycle, Pharmaceuticals, Medicines, Vaccines

## Abstract

**Introduction:**

Buyers of medicines and vaccines are increasingly interested in pooling their procurement to improve access to affordable and quality-assured health commodities. However, the academic literature has provided no detailed description of how pooled procurement mechanisms are set up and develop over time. These insights are valuable as it increases our understanding of implementing and operating pooled procurement mechanisms successfully. Therefore, the aim of this paper is twofold. First, to explore how such mechanisms evolve over time. Second, to clarify the work that is needed to set up and sustain a pooled procurement mechanism. These findings have been translated into our Pooled Procurement Guidance document.

**Methods:**

This qualitative study draws upon theoretical insights from organizational life cycles, collaborative and network governance, semi-structured interviews with procurement experts and academic and grey literature documents on pooled procurement of medicines and vaccines.

**Results:**

We identified four general developmental stages of pooled procurement mechanisms: promise, creation, early operational and mature. The promise stage is characterized by initiating engagement between participating actors, while they try to convert their perceived problem(s) or opportunities into a shared vision. The creation stage is where the participating actors formalize and design the mechanism through consensus-building, articulation of a shared plan, and mobilize resources to put the shared plan into action. The early operational stage is where the shared plan is being executed. The newly established or appointed procurement organization is required to learn fast from experience while showing flexibility to the changing needs of buyers and suppliers. Once operations are routinized, the mechanism enters the mature stage. During this stage, the pooled procurement organization develops into a trusted player that provides sufficient incentives for all actors involved. Importantly, pooled procurement mechanisms can stagnate or turn inactive at any time during the developmental process when alignment between actors is threatened.

**Conclusions:**

Pooled procurement mechanisms evolve over time. Setting up such mechanisms is a collaborative process that relies on intentional efforts by key actors involved. To increase the lifespan of pooled procurement mechanisms, key actors need to sustain a relative alignment of goals, needs, motivations and purpose of the mechanism throughout its entire life cycle.

**Supplementary Information:**

The online version contains supplementary material available at 10.1186/s40545-023-00574-9.

## Introduction

Pooled procurement, seen as a collaborative effort between buyers to consolidate their purchases, is implemented to achieve a variety of goals, including price reductions, improvement of procurement efficiency, incentivizing suppliers to secure supply and increase availability of products [1–3]. Although pooled procurement mechanisms have received increased attention as a potential solution to improve access to affordable and quality assured medicines, setting up such mechanisms is not a straightforward process in practice. Some pooled procurement mechanisms never get beyond their promise after years of discussions. Other mechanisms have launched, but have failed to achieve their intended goals, such as lowering prices. There are also examples of mechanisms that have been scaled down or even ceased to exist after a short period of operation [1, 4, 5].

In a recent review of the academic literature on pooled procurement mechanisms for medicines and vaccines [1], several essential elements were identified that appeared to be critical for setting up and operating pooled procurement mechanisms. These included compatible laws and regulations, sufficient technical capacity for accurate demand forecasting and financial capacity for buyers. Similarly, the pooled procurement organization needed sufficient budget to procure health products and cover organizational expenses, and technical capacity to carry out procurement. Suppliers needed sufficient incentives to participate, such as accurate demand forecasts, framework agreements, and a timely payment mechanism. The systematic review [1] also described the complexity and diversity in the operational models of pooled procurement mechanisms. These mechanisms varied in their structural form (ranging from a third-party organization that procures on behalf of its buyers to a more buyer’s owned/inter-buyer mechanism that operates more collaboratively), operational level (i.e., sub-national, national, inter-country and global level), type of products to be pooled (e.g., single source, single disease, single product type, multi-products) and motivations and goals of the pooled procurement mechanism (e.g., price reduction, increase availability, procurement efficiency and share technical capacity).

However, there are limited studies that explore how pooled procurement mechanisms came into existence and how they developed over time. Nollet and Beaulieu [6] explored the development of purchasing groups in the US healthcare sector, highlighting critical success factors and the need for adaptation to their changing environment. However, their study focused only on hospital collaborations and historical development of the entire “sector”, rather than a single collaboration initiative. Another recent study by Vogler et al. [3] on centralized national procurement in six European countries verified the lack of empirical insights into the development of pooled procurement mechanisms. Although the study provided rich descriptions of national level mechanisms, the authors emphasized the need for further study on pooled procurement mechanisms in inter-country settings. This paper aims to contribute to filling this gap.

To guide this empirical endeavour, we use theoretical insights from organizational life cycle literature, and collaborative and network governance, which we will expand upon in the following sections. These theoretical insights provide us with a more general understanding of the creation and development of networks, collaborations and organizations. This literature points us to the importance of looking at pooled procurement mechanisms not as a singular event, but as a process that evolves over time. Such an approach emphasizes the fact that these mechanisms require active effort by the actors involved to align the various motivations, goals and design of the mechanism. Insights from this literature can, therefore, help us to better understand what work is required to make pooled procurement mechanisms a success.

To our knowledge, no attempt has been made to take such a process-approach to describe and explore the development of pooled procurement mechanisms over time. Such insights are important as they contribute to the understanding of how pooled procurement mechanisms are formed and sustained while adapting to the evolving internal and external environment. Therefore, the aim of our is paper is twofold. First, to explore how pooled procurement mechanisms evolve over time. Second, to clarify what work and processes are needed within and between the various developmental stages of a pooled procurement mechanism. To reach this second aim, we translated the lessons learned into a Pooled Procurement Guidance document to help the development of pooled procurement mechanisms in practice. This study mainly focuses on buyer’s owned inter-country and global level pooled procurement mechanisms. However, we believe that insights of this study also apply to local and national level pooled procurement mechanisms.

## Theoretical background

To explore the development of and dynamics within pooled procurement mechanisms, we draw upon theoretical insights from literature on organizational life cycles and collaborative and network governance. The creation and functioning of collaboration initiatives is a widely discussed topic in the collaborative and network governance literature. In this paper, following Klijn and Koppenjan [7], collaborations and networks are used interchangeably. Ansell and Gash [8] pointed out that collaborations are characterized by extensive interaction between participating parties. These interactions foster joint action and mutual interdependence between parties while keeping a certain degree of autonomy. Collaborative governance studies often focus on the interaction processes and structures between the actors within those networks [9].

Pooled procurement has recently been defined as *“a collaboration initiative that consists of two or more buyers, or a third-party organization that procures on behalf of its participating members*” [1]. In theory, such a collaboration is characterized by high levels of interdependence, management and collective action between various public agencies (e.g., regulatory bodies, procurement agencies), governments (e.g., Ministry of Health, Ministry of Finance) and private parties (e.g., suppliers, distributors). To gain insight into how these collaborations develop and the work required to manage and sustain these interactions between actors we focus on different developmental stages.

### Developmental stages

Pooled procurement mechanisms are not static collaborations. They evolve during implementation and operation. Therefore, the elements and work required to form and sustain a mechanism depends on the stage of a mechanism. Using organizational life cycle theories allows us to better understand the processes that take place within and between each developmental stage of a pooled procurement mechanism. Since the 1960s, much has been written in the organizational life cycle literature on how organizations develop in largely predictable ways over time [10–14]. Although these analyses focus on different aspects of the organizational life cycle and apply the theory to different contexts, they all have certain general elements of the developmental process in common: the emergence, the growth and the maturity of the collaboration. Kenis & Provan [15] pointed out that the effort of newly emerging networks will mainly be directed towards “developing structures and processes”. When these are established, networks should focus on gaining legitimacy. Only after maturity has been reached, networks can be expected to operate efficiently and reach their predefined common goals.

#### Emergence

During the initial stages of emergence, participating actors need to be incentivized to engage with each other. Emerson et al. [9] refer to this as consequential incentives. These incentives can be either internal, based on problems, needs or opportunities, or external, based on a crisis or a threat. Other drivers for participation include the presence of a complex problem that cannot be solved independently [16] and interdependency of resources between participating actors [9, 17].

Although the boundaries can be fuzzy, Huxham and Vangen [18] have identified various categories and levels of aims that participating actors might have when joining a collaboration. They note that an actor is often not a single person, but can consist of multiple individuals representing departments or organisations with varying opinions and interests. Therefore, aims can be on the individual level, on the organizational level or on the collaboration level. Aims can also be externally driven, that is by actors outside the collaboration initiative. Another important distinction they make relevant to our paper, is that aims can be explicit, unstated or hidden. Although these distinctions can help understand and categorize aims of actors, the authors underline that aims are fluid. Multiple aims can be present simultaneously, they can interact and can also change over time.

Once actors have been incentivized for initial participation, actors need to interact in more systematic and deliberate ways to explore possibilities of collaboration. Emerson et al. [9] mentioned that these interactions, which they refer to as “principled engagement”, are characterized by four processes: discovery, definition, deliberation, and determination. The interaction starts with presenting “individual and shared interests, concerns and values” (discovery), followed by articulating agreed purposes, concepts, expectations, and assessment criteria (definition). Within deliberation, involved actors negotiate and try to resolve clashing interests and reach relative alignment [9]. To reach relative alignment, participating actors often need to go through a process called “fruitful conflict”. This process is characterized by actors that try to “enhance or advance knowledge, understanding, meaning, or capacity between different or opposing perspectives and interests” [19].

Finally, involved actors reach determinations, which include both substantive and procedural determinations [9]. This is the point, where the collaboration has to be formalized through agreements on its operations and organizational design [10].

During these various steps of interaction, actors need to develop professional and personal relationships. Emerson et al. [9] refer to this as shared motivation, including mutual trust, mutual understanding, internal legitimacy and commitment. These relationship-building efforts can be influenced by various factors, such as actors’ pre-existing and potentially differing (working) cultures, languages, procedures, customs, ideologies, history of collaboration, face-to-face dialogue, and continuity in representation [7, 9, 18, 20].

#### Growth

The growth stage is characterized by expansion and development of the collaboration initiative. Once personal relationships have been established and relative alignment on aims, goals and purpose have been achieved, the collaboration has to further define its operations and structure. This includes formalizing procedural and institutional arrangements, such as establishing an organizational structure with clear roles and responsibilities, a clear mandate, standardized and transparent procedures, fair allocation of benefits and no conflict of interest [9, 20, 21]. In addition, the collaboration needs to attract a sufficient pool of resources to start and gradually expand the operations. These resources include funding, personnel, expertise and time [9].

After the start of operations, the collaboration initiative will accumulate practical and operational knowledge. If knowledge and experience acquired during early operations can be monitored, evaluated and reflected on systematically, the collaboration can apply its outcome to optimize and diversify operations and specialize organizational structure further [10, 21]. This iterative process of learning from experience drives rapid expansion and growth of the collaboration initiative.

An important motivator to sustain commitment during the growth stage is the participating actor’s perceived benefit of the collaboration [14, 20]. This also includes the actor’s perceived benefits relative to the benefits of other participating actors. Schotanus et al. [22] refer to this as “fair allocation of gains”. If members do not experience this fair allocation, they might withdraw from the collaboration [14].

#### Maturity

During the maturity stage, the growth of the collaboration stabilizes and operations are routinized at optimal efficiency levels [13]. The outcomes and impact it has generated during the previous stage drives the collaboration to adapt to the changing internal and external environment to become sustainable [9].

Potential new members might also seek to join the collaboration in the maturity stage. These new members might bring new interests and goals. The collaboration needs to strike a balance between providing sufficient flexibility towards evolving and diverging interests while sustaining the predetermined goals and aims [21]. D’Aunno and Zuckerman [14] refer to this point as “critical crossroads”, while others refer to this as the transformation stage [10, 21]. If the collaboration does not react adequately to these changing dynamics and environment, the collaboration might evolve towards decline [13], where the progress might stagnate or even result in the collapse of the collaboration. However, this decline is not always linear. It can occur at any stage of the development, where alignment between members is threatened.

We use the stage-model to identify the processes of setting up pooled procurement mechanisms and explore the work that is required during the various developmental stages of pooled procurement mechanisms.

## Methods

### Study design

We conducted a multi-method qualitative study using a two-step study design. First, we developed Part 1 of the Pooled Procurement Guidance document (see Additional file 1 for the complete Guidance and Additional file 2 for Part 1 of the Pooled Procurement Guidance with data sources for the identified essential elements) by identifying essential elements for successfully implementing and operating a pooled procurement mechanism. A recent literature review of empirical papers on pooled procurement [1] served as the starting point for the development of our Guidance document. Based on the gaps that were identified in this review; we mobilized other sources of data to provide a more comprehensive overview of the elements that play a crucial role in pooled procurement mechanisms. We conducted a scan of academic and grey literature documents on pooled procurement. We scanned grey literature documents in various formats, such as feasibility studies, policy papers, reports, academic theses, presentations, and newspaper articles. These documents were identified through various sources including suggestions of key procurement experts included in our study, scanning reference lists of both academic and grey literature documents (i.e., snowballing), and targeted searches in online databases, such as WHO’s Institutional Repository for Information Sharing (IRIS), PubMed, Google Scholar, and various news outlets. We used the following search terms: pooled; bulk; joint; centralised and collaborative with procurement or purchasing in combination with medicine*; pharmaceutical*; drug*; vaccine*. We used Boolean operators to combine these search terms in the databases. Our search was not limited to a particular timespan to capture as many publications on pooled procurement mechanisms as possible. We ended our search in February 2023.

Second, to validate Part 1 of the Guidance document, we reached out to 27 purposefully selected procurement experts in several batches between November 2021 and May 2022 and asked them to comment on the Guidance. Their selection was based on their knowledge and expertise of the implementation and functioning of pooled procurement mechanisms. We identified these procurement experts from their publications and through our professional network on (pooled) procurement. We stopped our search for additional respondents after data saturation was reached. Of the 27 experts, 11 procurement experts returned their written feedback and suggestions.

We then invited these procurement experts to participate in a semi-structured interview to reflect on their feedback. During these interviews, we also asked respondents to further elaborate on *when* the processes, identified in Part 1 of the Guidance document, are essential. Our goal was to understand during which developmental stages these processes play a vital role. These insights were captured in Part 2 of the Guidance document. These semi-structured interviews were conducted virtually (e.g., Zoom, Microsoft Teams, and telephone) between December 2021 and May 2022, using an interview guide. We obtained oral and/or written informed consent for interviews and requested permission to audio-record them. Participants were anonymized using identification numbers that were stored separately from the study data. Interviews were conducted both in English and Dutch and lasted between 30 and 60 min. The participants were based on different parts of the world. The majority was based on Europe, with 1 participant from South America, 1 participant from the Middle East and 2 participants based on North America. Furthermore, 2 participants held an academic position (professor or associate professor) focusing on public procurement; 1 participant was a department director at a national public health research institute; 1 participant worked as a director at a research center on public procurement; 2 participants were director at an international pooled procurement organization, and 5 participants were procurement specialists and consultants with extensive international experience in the field of procurement and supply chain of medicines and vaccines. The background of the participants is summarized in Table 1.

**Table 1:** Category and number of participants in the study, *N* = 11

Category of participant	#
Procurement specialist/Consultant	5
Procurement agent	2
Academic	4

### Data analysis

The analysis of the expert opinions was an iterative process. For this, we used a constant comparative method approach [23]. As we collected our data in the form of written expert opinions, we compared and triangulated it with earlier collected data from the literature study, grey literature documents, theoretical insights, and previous interviews and expert opinion. This approach allowed us to verify our findings and take insights from previous data collection into account during subsequent data collection. During the data collection, we held multiple sessions within the research team until consensus was reached on the adaptation of the Guidance document.

For the purpose of this paper we analysed the semi-structured interview data using an abductive approach [24]. This approach, which is a recursive and reflexive process, allowed us to explore theoretical notions of organizational development and collaborations in the context of pooled procurement mechanisms while providing leeway to identify potential gaps and new insights that are relevant and unique to the development of pooled procurement mechanisms of medicines and vaccines. As a first step, the first author (KP) read the transcripts to familiarize with the content and coded the interviews. After this, the first author and at least one of the co-authors were involved in identifying relevant descriptive themes from the interview transcripts. Finally, we identified relevant analytical themes for each developmental stage of the pooled procurement mechanism, and we discussed these between all co-authors during several group meetings. Examples of such themes were stakeholder engagement, consensus-building between buyers, securing sufficient and predictable budget and creating sufficient supplier incentives. NVivo (12.7.0) was used as the qualitative data analysis software.

## Results

In this section, we present our Pooled Procurement Guidance document. The Guidance consists of two main parts. Part 1 of the Guidance identifies essential elements for each key actor in the pooled procurement mechanism. Part 2 of the Guidance explores the processes and work that are required to set up and sustain a pooled procurement mechanism during its various developmental stages. We present these different stages in the second part of our results section.

### Part 1: Essential elements for pooled procurement

We developed Part 1 of the Pooled Procurement Guidance document to provide a more comprehensive overview of the elements that play an essential role in the implementation and operation of pooled procurement mechanisms. We divided the actors into three groups of key actors: the buyers, the pooled procurement organization or secretariat, and the suppliers. We believe that these elements are often specific to a group of actors, but may differ slightly by type of procurement mechanism. For buyers, we differentiate between elements that are necessary for all buyers individually (even if procurement is outsourced to a third-party organization), and elements that are essential for buyers to share collectively, in a situation in which buyers participate directly in the management of the procurement mechanism, referred by us as a buyer’s mechanism.

The Guidance document also provides information about why each element is considered essential. For example, each participating buyer needs to have access to funding with which to buy medicines from the pooled procurement organization, even if that funding ultimately comes from an external donor. Without the capacity to allocate or attract funding, the buyer cannot procure through the mechanism, which holds true for both buyer’s mechanisms and third-party organization mechanisms. Meanwhile, all buyers collectively need to have a joint need for specific products to procure through the mechanism, including pack sizes, dosage forms and strengths. Without a joint need, pooling around specific types of products cannot take place, and therefore, buyers will lose the financial benefits resulting from economies of scale. Finally, shared cultural factors and values (e.g., language, traditions, etc.) among all buyers in a buyer’s mechanism is more likely to enhance trust among buyers and increase understanding of each other’s modus operandi and interactions.

However, the processes and types of work that are required to achieve some of these elements vary depending on the specific developmental stage of the pooled procurement mechanism. Therefore, we will continue zooming in on the processes and work, identified in Part 1 of the Guidance document, that are characteristic for each developmental stage.

### Part 2: Development of a pooled procurement mechanism

**Fig. 1:**
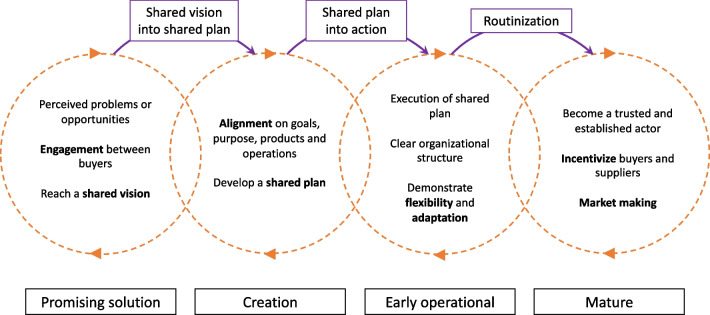
Schematic representation of the developmental stages of pooled procurement mechanisms

Drawing upon the theoretical insights on organizational life cycles and a review of the empirical literature on pooled procurement mechanisms [1], we have identified four ideal developmental stages: the promise stage, the creation stage, the early operational stage and the mature stage. The remainder of the results chapter is organized around these stages. Within each developmental stage, we highlight the essential elements that need to be present during each stage and the work that is required to reach the next stage. A schematic representation of these developmental stages and the work that is required to evolve between stages is provided in Fig. 1.

#### Promise stage

The first stage, which we refer to as the “promise stage”, is where each buyer (e.g., pharmacy, hospital, relevant authority in a district or country, etc.) decides that it is in their interest to participate in the development of a concrete plan for establishing a pooled procurement mechanism. A buyer, however, is often not a single person, but consists of multiple individuals, departments or organisations with varying opinions and interests. Therefore, achieving internal agreement within each buyer organization to participate should, therefore, not be neglected.

The goal in the promise stage is to create engagement between buyers in the pooled procurement mechanism by converting their perceived problem(s) or opportunities into a shared vision. To reach a shared vision, potential buyers need to engage with each other first. Motivations for buyers to engage might differ. Some buyers might perceive a problem for which pooled procurement provides a solution, while other buyers might see an opportunity that potentially improves their current situation. The recognition of a problem or opportunity might be initiated internally (i.e., from within the buyer), or externally (e.g., other buyers, global development organizations or intergovernmental organizations). Some buyers, however, might not necessarily experience an explicit problem or see a promising solution, but simply want to take part in the conversation or try something potentially new.

**Table:** 

**Box 1**. The role of individual actors in stakeholder engagement	
The Pan American Health Organization (PAHO) Revolving Fund (RF) is an example of a pooled procurement mechanism that has been initiated by an intergovernmental organization. PAHO, which is the regional office of the World Health Organization (WHO) in the Americas, adopted a resolution (CD25.R27) in 1977 to establish a revolving fund with the goal to improve immunization programs in the Americas by increasing access to affordable and quality-assured vaccines [25, 26]. The implementation of the PAHO RF in 1979 is often credited to the leadership and vision of a Brazilian epidemiologist, who was a strong advocate of reducing reliance on donor funding for vaccine procurement, adopting national immunization programs and one of the driving forces in the eradication of polio in the Americas [27–29]. This shows us that setting up such mechanisms also relies on the commitment and ownership of individuals in bringing together actors	

In addition to commitment and ownership, engaging actors in the promise stage is a long-term process that requires careful planning. Engagement can take place in various ways. Through informal relationships between buyers (e.g., health care organizations or pharmacies that procure certain health products jointly), through pre-existing political and structural relations or mechanisms (e.g., European Union countries procuring COVID-19 vaccines centrally), or initiated by a third-party.

**Table:** 

**Box 2**. Example of stakeholder engagement in faith-based organizations in Cameroon	
The implementation pooled procurement example of faith-based organizations (FBOs) in Cameroon was a result of regular engagement between stakeholders. This process consisted of the following steps: mapping of potential stakeholders; informing stakeholders about pooled procurement; identifying common goals, interests and barriers; adopting a shared vision and potential plan; and agreeing on a decision-making process. The Ecumenical Pharmaceutical Network, which is a non-profit Christian umbrella organization of faith-based healthcare organizations and professionals globally, played an active role in engaging potential actors. They intentionally organized meetings inviting all FBOs in Cameroon to discuss the possibility of setting up a pooled procurement mechanism [30]	

To reach a shared vision on pooled procurement during initial engagement, we identified several preconditions that have to be met: buyers’ motivation to participate should be compatible in terms of their relation to the proposed solution (i.e., the operational model of the pooled procurement mechanism), the potential benefits of buyers to participate in a pooled procurement mechanisms (e.g., price reduction, procurement efficiency, increased quality, sustainable supply, fair allocation of savings) should outweigh its costs (e.g., reduced autonomy, less flexibility), and buyers need to demonstrate willingness and ownership to collectively overcome their (potentially differing) problems.

**Table:** 

**Box 3**. Example of reaching a shared vision in the Organization of the Eastern Caribbean States	
The Organization of the Eastern Caribbean States Pharmaceutical Procurement Service (OECS/PPS) demonstrates how relatively similar sized islands managed to reach a shared vision during initial engagement [1]. Motivated by their shared problems of small market size, limited availability of essential medicines, fairly remote geographic location and limited financial and technical capacity, the OECS nations and the United States Agency for International Development (USAID) initiated discussions on pooled procurement of essential medicines [2, 31]. Pre-existing political and economic structures in the region, such as the OECS Secretariat and the Eastern Caribbean Central Bank, facilitated communication and high-level political commitment. As a result, participating OECS nations pledged a third of their pharmaceutical budget to the mechanism, even before the OECS/PPS was fully operational [31, 32]. These existing preconditions together with deep political commitment acted as a catalyst for OECS nations to agree on a shared vision: a buyer’s-owned mechanism that procures essential medicines for the public sector with the aim to achieve cost savings and higher procurement efficiency	

We can conclude from the above that the promise stage is essential for stakeholder engagement and reaching a shared vision between buyers. Once potential buyers have reached a shared vision, formed an initial working group to advance discussions and decided to develop a concrete plan for setting up a pooled procurement mechanism, the second stage of the development process begins.

#### Creation stage

The second stage, which we refer to as the “creation stage”, is the stage prior to the operations of the pooled procurement mechanism. The goal in this creation stage is to formalize the pooled procurement mechanism through articulation of the shared vision into a shared plan and to put the shared plan into action.

Often, developing a shared plan starts with a situational analysis of the buyers to determine the status quo, current needs and goals of the buyers [30, 33]. This is often carried out by an independent person or organization that is not related to the buyers. Based on this feasibility study, buyers need to go through a deliberation process during which buyers negotiate and try to resolve clashing interests. One respondent mentioned that this alignment process is often a negotiation process that benefits some actors more than others:

*“Often everybody benefits through pooled procurement, but generally some more than others. The question is, how big of a problem is that.”* [Academic]

In practice, the buyers need to transcend their individual goals and interests and reach an overarching consensus on the goals, purpose and operations of the pooled procurement mechanism [34]. This process is often led by an independent facilitator that is trusted by all involved parties [30]. During this process of consensus-building, several factors should be taken into account. Part 1 of our Guidance document shows that buyers should agree on factors such as determining the roles and responsibilities of actors, agreeing on financing of the mechanism, adopt laws, regulations and policies that allow for (international) pooled procurement, establish regulatory and policy harmonization between buyers and alignment on type of products to procure. A recently published WHO report mentioned that in the European context, the alignment of procurement timelines between buyers was another operational challenge that is often overlooked [35]. One respondent provided an example of buyers having different purposes for participating in a pooled procurement mechanism:

*Sometimes procurement takes place, and every now and then a few [hospitals] from the network participate. But a lot of people use it actually as a type of information exchange, and not necessarily for actual procurement. I notice this even in the Netherlands between homogeneous hospitals, who are part of different sorts of networks, but not procuring at all, while it is called a procurement collaboration.* [Academic]

These individual goals and motivations are influenced by the characteristics of each buyer, including market size, demographics, financial capacity, and bureaucratic structures. Relative homogeneity of characteristics between buyers related to their needs is an important facilitator for productive alignment. Diverging buyers’ characteristics are more likely to lead to diverging or even conflicting goals and motivations [1].

**Table:** 

**Box 4**. Example of diverging buyer characteristics in the Southern African Development Community	
The implementation of the Southern African Development Community (SADC) Pooled Procurement Services (SPPS), which dates back to the late 1990s, provides an illustrative example of diverging buyers’ characteristics [36]. Based on a situational analysis in the region [33], the 16 member states vary greatly on geographic, demographic, economic, pharmaceutical policy and procurement characteristics. From relatively remote island nations like Seychelles and Comoros, to landlocked countries such as Botswana and Zimbabwe, to large and populated countries such as DR Congo and South Africa. Similar disparities exist in characteristics such as economy in terms of GDP, health and pharmaceutical expenditure, burden of disease, availability of essential medicines, procurement and information systems, and medicine regulation. These wide variations among member states are highly likely to affect the incentives and motivations of each member state to participate in SPPS. Although the SADC Health Ministers approved the SADC Strategy back in 2012 [37], suggesting a stepwise approach starting with information and work sharing, the challenges in operationalizing SPPS highlight the complexity of aligning diverging characteristics, goals and motivations	

Factors that could facilitate the alignment of goals and motivations in more homogenous buyer’s mechanisms include open communication and continuity in representation, transparent data and information sharing on factors, such as suppliers, prices and demand planning, shared cultural factors and values, and trust between buyers, including no history of failed collaborations. One respondent mentioned that trust consists of various dimensions:

*“There are different types of trust: competence, honesty, and benevolence. If the buyers don’t trust each other, a third-party can play an important role in data gathering.”* [Academic]

Another respondent underlined that trust does not always have to be present between all layers of an organization or individuals to engage:

*If it is like a top-management decision that we should collaborate, and the trust exists between the top-management, it doesn’t necessarily have to translate to the trust and relationship between the operational levels. (…) I have a feeling that if the benefit or the problem is big enough, or if there is a strategic decision, they have to do it.* [Academic]

After alignment on goals, motivations and operations has been created, the pooled procurement organization or secretariat, which carries out the actual procurement, needs to be appointed or established. The structure of the pooled procurement organization depends on the structural form of the mechanism. This structural form ranges from a third-party organization procuring on behalf of its buyers, to a more buyer’s owned mechanism that operates more collaboratively [1]. Examples of existing pooled procurement mechanisms show that third-party organization pooled procurement mechanisms are often led by an organization that operates independently from its buyers. Examples of such organization include the Global Drug Facility (GDF), PEPFAR and hospital group purchasing organizations in the United States.

There are also buyer’s owned mechanisms, which are more collaborative in nature. These are generally governed by three main types of pooled procurement organizations:

1. Lead buying organizations, in which the responsibility of operations is outsourced to one buyer in the collaboration [38]. One example is Tanzania’s Medical Stores Department in the newly established SADC pooled procurement mechanism [39].
2. Shared-responsibility organizations, in which buyers share a fairly equal distribution of tasks. Examples include the Gulf Health Council for the Gulf Joint Procurement mechanism and the secretariat of the Organisation of Eastern Caribbean States (OECS).
3. A more hybrid governance approach, which can be classified as ‘rotating secretariat’, is an organization form in which the responsibility of the operations rotates between buyers in predetermined time intervals. The Baltic Procurement Initiative is an example of this [35].

The Pacific Island Countries provide an illustrative example of a failed pooled procurement mechanism, where buyers could not agree on some of the abovementioned preconditions. Discussions on pooled procurement came to a halt in the creation stage due to the lack of a combination of factors such as no harmonized rules and regulations, a failure to finance and establish a dedicated procurement secretariat, cultural differences and a lack of trust between countries [5, 40].

To realize any of the buyer-owned mechanisms, buyers need to mobilize resources to put the shared plan into action. These resources consist of securing sufficient, timely and predictable budget both for procurement and to cover organizational expenses, hiring staff that is sufficient in numbers and expertise, and providing physical and technological infrastructure to facilitate operations and communication.

The findings show us that the creation stage is crucial for consensus-building and reaching relative alignment between buyers on goals, purpose, products and operations of the pooled procurement mechanism. In addition, buyers need to develop a shared plan, in which buyers formalize the roles and responsibilities of actors and the pooled procurement secretariat. Once the buyers have put their shared plan into action by setting up or appointing a pooled procurement organization with sufficient resources, staff and expertise, the third stage of the development process begins.

#### Early operational stage

The third stage, which we refer to as the “early operational stage”, is where the pooled procurement organization has been launched and starts procuring their first products. The goal in this stage is to execute the shared plan into shared practice. After establishing or appointing the pooled procurement organization or secretariat in the creation stage, several organizational elements should be taken into consideration to facilitate the efficient functioning of the organization. These include the presence of an organizational and good governance structure with clear roles and responsibilities, a clear mandate, standardized and transparent procedures, and no conflict of interest. One procurement expert added that buyers need sufficient representation in the pooled procurement organization to guide and oversee its operations. Another respondent pointed out that these elements are not necessarily essential, but act as facilitators:

*Is it really essential? Or is it something that they firefight as they come across it? I don’t know. (...) In the limited cases I have seen, I don’t always see this. I see it being considered as something very important. After they go into it, they say: “oh, we should’ve thought about this from the beginning. We should have had this clear mandate. We should have had clear procedures.”* [Academic]

The sustainability of the mechanism will greatly depend on the adaptability and flexibility of the pooled procurement organization to overcome operational problems during this stage. These problems might include attracting and hiring dedicated and qualified staff, collect timely payments from buyers, adherence of the buyers to procure through the mechanism, achieving favourable contract conditions from suppliers, and carrying out accurate demand forecasting based on reliable data. Several respondents underlined the issue of inaccurate demand forecasting as one of the biggest challenges:

*The other challenge is also the data. Because you need to have near accurate forecasts. To be able to tell the manufacturers: “look, in the next year or two, this is what we are looking at.” But you don’t have the data to inform a very good quantification. So that is the very huge challenge that we noticed most countries are facing.* [Procurement expert]

Another respondent provided a specific example of inaccurate demand forecasting in an inter-country pooled procurement mechanism:

*There is a gap between primary initial quantity and the final quantity procured after the announcement of the final award. And sometimes they [i.e., countries] submit 1 million tablets for an item to receive the price. And after the award, they buy about 5 million. This inaccuracy in planning is a big challenge in procurement.* [Procurement agent]

A feasibility study on the SADC pooled procurement mechanism provides us insights into other challenges that pooled procurement mechanisms might face in the early operational phase. These challenges include a lack of regulatory harmonization, limited political commitment, different product needs and procurement goals generated by divergent characteristics (e.g., geography, demography, economy, pharmaceutical policy, procurement processes, etc.), a lack of an efficient payment mechanism that allows for upfront payments, and laws and regulations that limit international pooled procurement [5, 41].

During the early operational stage, suppliers also need to be invited and incentivized to participate in the mechanism. If suppliers do not experience sufficient incentives to participate, procurements can fail. This can be caused by a lack of supplier interest or by non-compliance to the terms and conditions set by the pooled procurement organization [3]. This can have far-reaching consequences, particularly in the early operational phase, where buyer–supplier relationships have not been well-developed yet and alternative procurement channels are limited. One respondent mentioned that incentivizing suppliers often starts with knowing your suppliers:

*You need to have a sufficient number of suppliers, but also to know your suppliers. So, for me it makes sense to have some prequalification of the suppliers, which in some countries is really just a formality. They just give you the name and the number of the company and I think that doesn’t work. I would also be in favour if suppliers, who used to fail or to commit that they are, for instance, then blocked for some time, or at least blacklisted*. [Academic]

Our findings show that the sustainability of the pooled procurement mechanism relies on the flexibility and adaptability of the pooled procurement organization to overcome initial problems during the early operational stage. Once the pooled procurement organization has overcome these operational problems, their work has been routinized, the suppliers are willing to participate and the buyers have reconfirmed that the value of pooled procurement outweighs its costs, the mechanism enters the fourth stage.

#### Mature stage

The fourth stage, which we refer to as the “mature stage”, is where the pooled procurement organization has become an experienced and reputable actor that is recognized by other actors in and closely related to the pooled procurement mechanism. The goal in this mature stage is to develop the mechanism into a sustainable practice. Reinforced by a positive track record and reputation, the pooled procurement organization can also start providing incentives to suppliers for products that are demanded by its buyer, but were not financially attractive or feasible before to produce or supply (i.e., market shaping). This is often seen around disease- or product-specific third-party pooled procurement mechanisms that focus on a limited set of products. One such example is UNICEF’s vaccine procurement mechanism. The vaccine market is generally characterized by high supplier concentration because of high costs and complexity of manufacturing [42]. Therefore, when UNICEF faced shortages of supply and suppliers of certain vaccines, it shifted its vaccine procurement strategy. As a reaction to these shortages and associated price volatilities, UNICEF’s focus changed from “high-volume-low-price” to a more “healthy market” oriented approach [43]. They achieved this healthy market principle, in which supply and demand are more balanced, mainly through a combination of accurate demand forecasting, long-term agreements, and multiple-winner awards. These market-shaping efforts were confirmed by one of the respondents:

“*After they got into the situation where the market got too concentrated and they actually lost supply and technical capacity of vaccines because some of the players were not interested in the vaccines, they used part of their demand to shape the market like what the WHO does, for example. To share technical capability with the countries. And they also [promised] these future contracts by saying: “We will give you the technical capability, the knowledge, we will even send someone from the WHO or from UNICEF to sit down with you about the production. And if you manage to get the right quality and the capacity, then we will buy from you.”* [Academic]

Another important incentive to maintain the commitment of both buyers and suppliers is the user-friendliness and the positive reputation of the pooled procurement organization. This positive reputation is reinforced by providing a rounded procurement service adding financial and operational value, including capacity building, risk sharing, flexibility, responsiveness, e-procurement, and transparency.

**Table:** 

**Box 5.** Example of user-friendly and rounded procurement services by PAHO Revolving Fund (RF)	
Many of these rounded procurement services have been underlying the success of the PAHO RF. In addition to pooling demand, central contracting and dedicated staff, the PAHO Secretariat also provides technical, financial and organizational incentives to both buyers and suppliers. It supports buyers in areas such as accurate demand planning, harmonizing legislation, advocating for national budget lines, implementation of national immunization programs, financial flexibility by allowing buyers to pay after the receipt of goods, and prequalification of suppliers [5, 25, 44–46]	
At the same time, the PAHO Secretariat incentivizes suppliers by providing access to a consolidated and sustainable market, financial security through a predictable, timely and efficient payment mechanism and long-term framework agreements, reduced transaction costs and operational efficiency through standardization of products and processes [42, 45]	

We can conclude that the pooled procurement organization develops into the mature stage once operations are routinized and the procurement organization has become a trusted and reputable actor that provides long-term incentives for both buyers and suppliers in the mechanism.

#### Developmental stages of a pooled procurement mechanism

Based on our analysis of the theoretical literature, grey literature documents, and insights provided by our respondents, we have identified four general stages of a pooled procurement mechanism: the promise stage, the creation stage, the early operational stage, and the mature stage. These stages are presented in Table 2 with their corresponding main goal to be achieved during each stage.

**Table 2:** Developmental stages of a pooled procurement mechanism

Developmental stage	Main goal
Stage 1: Promise stage	To create engagement between participating actors and to convert the perceived problem(s) or opportunities into a shared vision
Stage 2: Creation stage	To formalize the pooled procurement mechanism through articulation of the shared vision into a shared plan and put the shared plan into action
Stage 3: Early operational stage	To execute the shared plan into shared practice
Stage 4: Mature stage	To develop the mechanism into a sustainable practice

In addition, we have developed a schematic representation of these developmental stages in Fig. 1. This representation includes the building blocks that need to be present within each stage, and the work that is required to reach the next stage. The development of the stages is presented as cyclical to depict the non-linearity of the work that is required to reach from one stage to the other.

#### Fragility of alignment

Even though the stages in Fig. 1 are presented cyclical, our analysis might give the impression that the development of a pooled procurement mechanism progresses somewhat linearly. However, when alignment between actors is threatened, pooled procurement mechanisms can also struggle to sustain or develop further, which happens more often than not. This is when the mechanism turns inactive, falls back to earlier stages, transforms into a new model or stops existing. Like the development process, this process of decline is often not linear and it does not chronologically follow certain stages. Therefore, it should not be seen as a separate stage. It can happen at and between any stage of its lifecycle, where alignment between members is threatened. Many mechanisms have been dreamed of but never born, while others experience excessively long creation periods and then cease to exist perinatally.

ACAME (African Association of Central Medical Stores for Essential Drugs) is an example of an inter-country pooled procurement mechanism that was discontinued after its initial pilot. After the devaluation of the CFA franc in the early 90s, several central medical stores of francophone countries in the Western African region established ACAME in 1996 with the goal to improve access to affordable and quality-assured medicines and to increase information sharing among participating members [5, 47, 48]. In 1998, Guinea, Mali and Niger participated in a pilot to jointly procure 5 antimicrobials. Although this pilot resulted in financial savings for each country between 7% and 27%, the project was discontinued due to a lack of political commitment [5]. Challenges reported during its operational period included political instability in the region, a lack of procedural transparency, delayed deliveries, challenges with product registration and no unified payment mechanism [47, 49]. Since then, ACAME has continued as an information-sharing platform on areas such as pharmaceutical pricing, availability, quality, technical specifications, and promotion of regulatory harmonization between participating countries [5, 50].

The Gulf Cooperation Council’s (GCC) pooled procurement mechanism is another example that shows that alignment between buyers is a dynamic process, which has to be sustained during the entire lifecycle of the mechanism. The Gulf Joint Procurement program, which is carried out by the Gulf Health Council (GHC), was established in 1978 as one of the first inter-country collaboration initiatives on pooled procurement. It was set up with the goal to consolidate relations and strengthen integration between member states, and promote health for all its citizens in the six Member States [25, 44]. Although the GCC countries managed to create relative alignment on the goals, purpose and operations of the mechanism initially, the current transition in some of the GCC countries towards national procurement threatens the sustainability of the mechanism. One procurement agent explained that Saudi Arabia, accounting for over 70% of the market size in the GCC, recently started procuring their medicines through the national centralized procurement agency (i.e., NUPCO) to reduce duplication of work, increase autonomy and spending efficiency. As a result, the total volume and value of health products procured through the GCC Joint Procurement Program has reduced significantly:

“*Previously, we procured around 2.8 billion dollars for all GCC countries. Currently around 1.5 or 1.7 billion dollars.*” [Procurement agent]

These examples underline that pooled procurement mechanisms that are unsuccessful in sustaining alignment or reacting adequately to the changing internal and external environment are vulnerable to stagnation, fall back to earlier stages, develop into something new, or might even cease to exist.

## Discussion

The aim of this study was to analyse the development of pooled procurement mechanisms over time and to provide a clear understanding of the work and processes required for their success. Our analysis shows that setting up a pooled procurement mechanism often includes long-term processes that generally evolve along the lines of four developmental stages: the promise stage, the creation stage, the early operational stage and the mature stage. Although strongly interconnected, we have subdivided the emergence phase, identified in our theoretical background, into a “promise stage” and a “creation stage”. We believe that this distinction allowed for a more comprehensive examination of the processes that are required to evolve from a shared vision (i.e., promise stage) into a shared plan (i.e., creation stage). During this development, involved key actors have to gradually shift their focus from an individual to a collective perspective. This means that after initial engagement between key actors and internal deliberation on potential benefits and costs, buyers need to transcend their individual goals and interest to reach alignment on collective goals.

Applying collaborative governance and life cycle theories to the context of pooled procurement mechanisms has provided us insight into the general development of a pooled procurement mechanism. Various examples of inter-country pooled procurement mechanisms show that initiating, building consensus, and maintaining pooled procurement mechanisms is a complex and laborious process, that should not be underestimated. In addition, our findings underline that alignment between key actors is fragile. Alignment is a reflexive and recursive process that should be sustained during the entire life cycle of a mechanism. Even after the mechanism has reached the mature stage.

In this paper, we have mainly explored the development of buyer’s owned or inter-buyer pooled procurement mechanisms. Pooled procurement mechanisms of third-party organizations, however, appear to develop differently. Although third-party organizations were not the main focus of this study, we believe that further research to increase our understanding of the development of such organizations will provide significant scientific and practical value for the implementation and operation of pooled procurement mechanisms. In contrast to inter-buyer mechanisms, third-party pooled procurement mechanisms tend to be centred around specific diseases (e.g., HIV, TB, Malaria) or products (e.g., vaccines); operate and serve buyers on a global level; have limited involvement of buyers in its operations, governance and decision-making processes; require less harmonization and consensus-building during implementation; are potentially setup with public–private partnerships; and lack a guaranteed market to sell their products, since buyers are generally not the initiators of such mechanisms [1, 2, 51, 52]. Understanding these differences in characteristics are relevant, since inter-buyer pooled procurement mechanisms have often been promoted based on the successes of these global health organizations in terms of consolidating demand and reducing prices [1]. However, there have been no detailed studies to date identifying and setting out these differences in characteristics between these varying structural forms. Therefore, we plan further studies to explore the characteristics and development of third-party organization pooled procurement mechanisms, and compare these findings with inter-buyer pooled procurement mechanisms.

### The purpose of the Pooled Procurement Guidance document

Earlier academic studies [1, 2] have attempted to describe what factors play a role in the functioning of pooled procurement mechanisms. Although these studies identified factors that are relevant for the functioning of particular pooled procurement mechanisms, they have not attempted to combine these factors into a general framework. We believe that policy makers and procurement experts can benefit from a more comprehensive overview of how pooled procurement mechanisms develop over time and the elements that play an essential role in setting up and functioning of such mechanisms.

Our Guidance highlights that setting up a pooled procurement mechanism is a complex process that requires consideration of multiple components by each key actor involved (e.g., buyers, procurement organizations, and suppliers). However, it is important to keep in mind that this Guidance document is not meant to be treated as a finished product that universally applies to all pooled procurement mechanisms in its current form. As our analysis shows, creating a successful pooled procurement mechanism requires conscious effort and collaboration from all key actors involved. Our guidance document should be used as a compass with essential elements to consider, rather than a strict roadmap with a checklist to follow.

We believe that practical application and adaptation to real-life scenarios will greatly enhance the usefulness of this Guidance document in improving existing or developing new pooled procurement mechanisms. Some elements might be more relevant to consider in specific contexts, compared to others. For example, a lack of regulatory harmonization between buyers might be a dealbreaker for procurement on inter-country level, but might be less relevant to consider when pooling takes place between hospitals within the same country. Another point for consideration is that the Guidance document specifies what elements are relevant for which key actor, but not during which developmental stage. Some elements, such as trust among buyers, might be essential to sustain during all stages, while other elements, such as a clear mandate for the organization, might be relevant from the early operational stage onwards. Future applications and in-depth case studies using this Guidance document are needed to further clarify our understanding of when, how and why certain elements are essential under which specific circumstances.

### Limitations

Our study has some potential limitations. The number of key procurement experts included in our study as respondents might carry a risk of bias. However, triangulation of our data sources (e.g., theoretical insights from organizational life cycles, collaborative and network governance, systematic review of academic literature [1], grey literature documents, and semi-structured interviews), and triangulation of our analysis by working within a research team, increases our confidence in the validity and reliability of our study findings. Furthermore, both parts of our Pooled Procurement Guidance document might benefit from practical application. This can be in the form of in-depth or comparative case studies of existing and/or failed pooled procurement mechanisms.

## Conclusion

This article provides a comprehensive overview of the process of setting up a successful pooled procurement mechanism for medicines and vaccines. It shows that pooled procurement mechanisms are long-term collaborative processes that develop over time. Understanding the developmental path of pooled procurement mechanisms is crucial to increase the chances of successful implementation and functioning. Our Pooled Procurement Guidance document contributes to this understanding in three ways: it provides a comprehensive overview of essential elements for each key actor in the mechanism to consider; it explores and describes the four general developmental stages of pooled procurement mechanisms; and it clarifies the work that is required to form and sustain such mechanisms. We believe that alignment between key actors on goals, needs, motivations and purpose of the pooled procurement mechanism is a dynamic and reflexive process. For pooled procurement mechanisms to survive, this relative alignment has to be sustained during the entire lifecycle of the mechanism and requires continuous work and reflection. The elements identified in the different stages in this paper can help to direct this work.

## Supplementary Information

**Additional file 1.** Pooled Procurement Guidance.

**Additional file 2.** Part 1 of Pooled Procureent Guidance with data sources.

## Data Availability

The interview data generated and/or analyzed during the current research are not publicly available as individual privacy could be compromised. All other data is available in the public domain.

## Abbreviations

ACAME: African Association of Central Medical Stores for Essential Drugs
FBOs: Faith-based Organizations
GCC: Gulf Cooperation Council
GDF: Global Drug Facility
GHC: Gulf Health Council
IRIS: Institutional Repository for Information Sharing
NUPCO: National Unified Procurement Company
OECS/PPS: Organisation of the Eastern Caribbean States Pharmaceutical Procurement Service
PAHO RF: The Pan American Health Organization Revolving Fund
PEPFAR: President’s Emergency Plan for AIDS Relief
SADC: Southern African Development Community
SPPS: SADC Pooled Procurement Services
UNICEF: United Nations Children’s Fund
USAID: United States Agency for International Development
WHO: World Health Organization
