# Supplementary material for: From promise to practice: a guide to developing pooled procurement mechanisms for medicines and vaccines
Source: J Pharm Policy Pract. 2023 Jun 14;16:73. doi: 10.1186/s40545-023-00574-9 (PMC10264874; doi:10.1186/s40545-023-00574-9)
Supplement: Supplementary file 1 — Additional file 1. Pooled Procurement Guidance. [file 40545_2023_574_MOESM1_ESM.pdf]

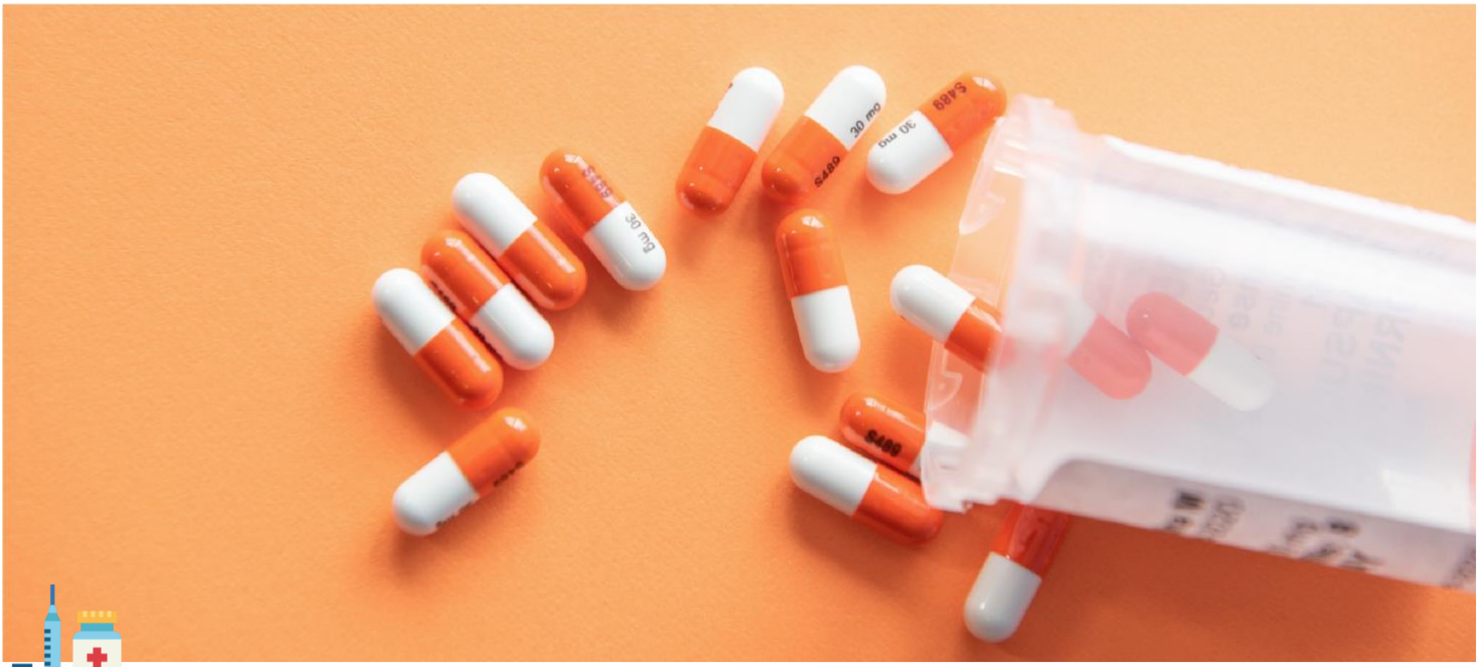

Pooled Procurement Guidance

Part 1: Essential Elements of Pooled Procurement

Part 2: Developmental Stages of Pooled Procurement Mechanisms

---

Koray Parmaksiz, Maarten Kok, Hester van de Bovenkamp, Roland Bal

Erasmus School of Health Policy & Management

Purpose of the Guidance

This Pooled Procurement Guidance was developed with the purpose to provide a more comprehensive overview of the elements that play an essential role in setting up and operating pooled procurement mechanisms (Part 1) and how such mechanisms develop over time (Part 2).

This Pooled Procurement Guidance is intended to provide practical guidance for academic researchers, policy makers and others as they explore, research or prepare to implement a pooled procurement mechanism. The contents of this Pooled Procurement Guidance might not universally apply to all pooled procurement mechanisms in its current form. Some elements might be more relevant to consider in specific contexts, compared to other elements. Therefore, this Guidance should be interpreted, used and applied as a guiding compass, rather than a defined roadmap.

Please send any further feedback, questions or requests to **korayparmaksiz@gmail.com**

PART 1: ESSENTIAL ELEMENTS OF POOLED PROCUREMENT

A. BUYERS

a) All buyers need to have individually:

**Essential elements**	**Explanation**
1. Perceived problem for which pooled procurement may be a solution	For a buyer to participate in a pooled procurement mechanism, the buyer needs to experience a problem for which pooled procurement might provide a solution, or see an opportunity that might potentially improve their current situation.
2. Motivations that outweigh the opportunity costs	In addition to the problem, the buyer needs to perceive that the benefits of participation (e.g., price reduction, procurement efficiency, increased quality, sustainable supply) will outweigh the costs. Some factors that contribute to the buyer's motivation are the user-friendliness of the platform, degree of shared-decision making, flexibility in participation, and responsiveness of the pooled procurement organization.
3. Budget, either internal or external (through donors)	The buyer needs to have or be able to attract sufficient budget, either through internal budget or externally through donors
4. Sufficient technical capacity (e.g., demand forecasting)	The buyer needs to have sufficient technical capacity to participate in a pooled procurement mechanism. For example, it needs the capacity to carry out accurate demand forecasting to procure the accurate number of products.
5. Compatible laws, regulations and policies that allow for (international) pooled procurement	The buyer needs laws, regulations and policies in place that allow for procurement, import and regulatory harmonization (e.g., patent laws, import tariffs, willingness to accept joint product approval, etc.) in (international) pooled procurement mechanisms.

b) If buyer's mechanism, all buyers combined, need to have:

**Essential elements**	**Explanation**
6. Demonstrated willingness to solve their problem collectively through pooled procurement (shared vision)	The buyers need to demonstrate the willingness to solve their problem(s) collectively through a pooled procurement mechanism. This willingness includes political will, leadership and ownership by relevant individuals or organizations.
7. Alignment on goals, purpose and operations of the pooled procurement mechanism (shared plan)	The buyers need to align on goals, purpose and operations of the pooled procurement mechanism. This does not necessarily mean that all buyers need to have the same goals, purpose and operations for the mechanism. As long as they are not conflicting.

8. Joint need for specific products (product alignment)	The buyers need to have a joint need for specific products. If there is no joint need, pooling around specific type of products cannot take place, and therefore buyers will lose the financial benefits resulting from economies of scale.
9. Sufficient market size	The buyers combined need to have a sufficient market size to attract suppliers for a favourable price.
10. Sufficient and stable financial capacity	The buyers combined need to have sufficient and stable financial capacity to procure through the pooled procurement mechanism.
11. Regulatory harmonization (e.g., shared quality standards, joint assessment, mutual recognition, etc.)	Sellers are potentially attracted to pooled procurement systems because it increases their market size while reducing the need to reregister products in each country, an expensive and time-consuming proposition. If there is no shared system for approving products and allowing access to markets, then this advantage is lost in practice.
12. Trust (in other buyers and the pooled procurement organization)	Buyers need to reach a certain level of trust in each other and the pooled procurement organization for them to share data and allocate budget. Trust levels can grow over time and can be reinforced by positive experiences with the pooled procurement mechanism.
13. Transparent data and information sharing	To reduce information asymmetry, the buyers need to have a mechanism in place that allows for transparent data and information sharing on suppliers, prices and demand forecasts between each other. A lack of transparency will negatively affect trust between buyers, and will result in the pooled procurement mechanism not reaching its full potential.
14. No history of conflict or failed collaboration	The level of trust between buyers might be negatively affected if the buyers share a history of conflict or a history of failed collaboration.
15. Homogeneity of buyer's characteristics related to their needs	Buyers need to share similar characteristics (e.g., market size, demographics, financial capacity, bureaucratic structures, etc.) related to their collective needs (e.g., type of products, motivations, goals, etc.). If there is no homogeneity between buyers related to their needs, there is a greater possibility of conflicting interests within the pooled procurement mechanism, which might negatively affect the sustainability of the mechanism.
16. Shared cultural factors and values (e.g., language, traditions, etc.)	Buyers sharing similar cultural factors and values (e.g., language, traditions, etc.) are more likely to trust and understand each other and their way of working/interacting, which will benefit the pooled procurement mechanism.
17. Existing political or structural mechanisms	Buyers having pre-existing political or structural mechanisms in place are more likely to trust and understand each other and their way of working/interacting. These pre-existing political or structural mechanisms do not have to be limited to the area of medicine procurement. A greater level of interdependence between buyers to solve their problem(s) in other areas will stimulate buyers' collaboration and adherence to the mechanism.

B. POOLED PROCUREMENT ORGANIZATION

Essential elements	Explanation
1. Organizational and good governance structure with clear roles and responsibilities	The pooled procurement organization needs an organizational and good governance structure with clear roles and responsibilities that buyers and suppliers trust to do business with
2. Clear mandate	The pooled procurement organization needs a clear mandate that is provided by the buyers on whose behalf they procure
3. Standardized and transparent procedures	The pooled procurement organization needs standardized and transparent procurement procedures to increase trust and user-friendliness
4. Sufficient, predictable and timely budget, either internal (through service fees) or external (through donors) to carry out pooled procurement	The pooled procurement organization needs sufficient, predictable and timely budget to procure medicine, to attract suppliers, and to respond to unforeseen circumstances with sufficient financial buffers.
5. Sufficient, predictable and timely budget, either internal (through service fees) or external (through donors) to cover organizational expenses	The pooled procurement organization needs sufficient, predictable and timely budget to cover their organizational expenses, including salaries, insurances and rental leases.
6. Predictable, timely and efficient payment mechanism	Sellers will be more attracted to the pooled procurement mechanism if the pooled procurement organization has a predictable, timely and efficient payment mechanism, including single source payment, single currency, acceptable payment period, upfront payment.
7. Human resources (sufficient in numbers and expertise)	The pooled procurement organization needs sufficient and expert human resources. This also means that the pooled procurement organization needs sufficient budget to provide competitive salaries to attract skilful staff.
8. Sufficient technical capacity (e.g., procurement, quality assessment, forecasting, etc.)	The pooled procurement organization needs sufficient technical capacity to carry out tenders, to assess quality of products, to aggregate demand data, to provide capacity building for buyers, etc.
9. Positive reputation	The pooled procurement organization needs to develop a positive reputation, which is based on trust from other actors in the pooled procurement mechanism. Positive reputation is necessary to attract and be entrusted with funding from buyers and funders. This positive reputation is reinforced by providing a rounded procurement service to its buyers and suppliers, including capacity building, risk sharing, market shaping, responsiveness, accountability and transparency.
10. No conflict of interest	The staff at the pooled procurement organization should have no conflict of interest. The organization should operate independently, maximizing the benefits for all its buyers.
11. User-friendliness (both towards buyers and sellers)	The pooled procurement organization should provide services to buyers and suppliers in a user-friendly manner with a reliable management information system. The benefits of procuring and supplying through the pooled procurement organization should outweigh the costs, both in terms of finances and effort.

C. SUPPLIERS

Essential elements	Explanation
1. Sufficient number of qualified suppliers	In the case of generic medicines, an effective pooled procurement mechanism needs a sufficient number of qualified suppliers in the market to increase supply security. A potential way of increasing the number of qualified suppliers is for the pooled procurement organization to incentive suppliers for production and supply.
2. Sufficient production incentives	Incentives for suppliers to produce products for the pooled procurement organization include a consolidated and sufficient market size; warehouse(s) providing buffer stock; take-off agreements; long-term framework agreements; multiple-buyer tenders.
3. Sufficient supply incentives	Incentives for suppliers to supply/sell products to the pooled procurement organization include predictable timely and efficient payment mechanism; regulatory harmonization; user-friendliness; positive reputation.
4. Sufficient number of distributors with favorable delivery terms	An effective pooled procurement mechanism needs a sufficient number of distributors/logistics companies that are willing to deliver the product(s) for favourable delivery terms (including lead time, costs, incoterms).

DEVELOPMENTAL STAGES

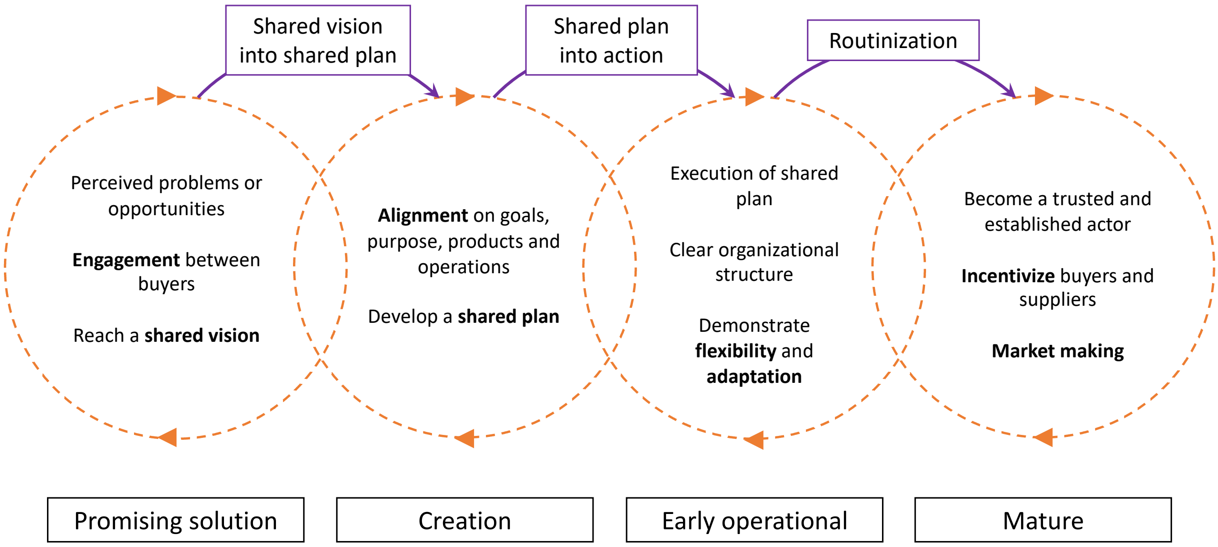

Stage 1 – Promise stage: The main goal is to create engagement between participating actors and to convert the perceived problem(s) or opportunities into a shared vision.

Stage 2 – Creation stage: The main goal is to formalize the pooled procurement mechanism through articulation of the shared vision into a shared plan and put the shared plan into action.

Stage 3 – Early operational stage: The main goal is to execute the shared plan into shared practice.

Stage 4 – Mature stage: The main goal is to develop the mechanism into a sustainable practice.
