# Supplementary material for: From promise to practice: a guide to developing pooled procurement mechanisms for medicines and vaccines
Source: J Pharm Policy Pract. 2023 Jun 14;16:73. doi: 10.1186/s40545-023-00574-9 (PMC10264874; doi:10.1186/s40545-023-00574-9)
Supplement: Supplementary file 2 — Additional file 2. Part 1 of Pooled Procureent Guidance with data sources. [file 40545_2023_574_MOESM2_ESM.docx]

| **Essential elements/actor** | **Explanation** | **References** |
| --- | --- | --- |
| 1. **Buyers** |  |  |
| **All buyers need to have individually** (both for buyers in buyer's PPM & third-party organization PPM): | |  |
| 1. Perceived problem for which pooled procurement may be a solution | *For a buyer to participate in a pooled procurement mechanism, the buyer needs to experience a problem for which pooled procurement might provide a solution, or see an opportunity that might potentially improve their current situation.* | *(1,2)* |
| 1. Motivations that outweigh the opportunity costs | *In addition to the problem, the buyer needs to perceive that the benefits of participation (e.g., price reduction, procurement efficiency, increased quality, sustainable supply) will outweigh the costs. Some factors that contribute to the buyer’s motivation are the user-friendliness of the platform, degree of shared-decision making, flexibility in participation, and responsiveness of the pooled procurement organization.* | (1,3–6) |
| 1. Budget, either internal or external (through donors) | *The buyer needs to have or be able to attract sufficient budget, either through internal budget or externally through donors* | *(7,8)* |
| 1. Sufficient technical capacity (e.g., demand forecasting) | *The buyer needs to have sufficient technical capacity to participate in a pooled procurement mechanism. For example, it needs the capacity to carry out accurate demand forecasting to procure the accurate number of products.* | (6–10) |
| 1. Compatible laws, regulations and policies that allow for (international) pooled procurement | *The buyer needs laws, regulations and policies in place that allow for procurement, import and regulatory harmonization (e.g., patent laws, import tariffs, willingness to accept joint product approval, etc.) in (international) pooled procurement mechanisms.* | (6,8,10–13) |
| **If buyer's mechanism, all buyers combined, need to have:** | |  |
| 1. Demonstrated willingness to solve their problem collectively through pooled procurement (shared vision) | *The buyers need to demonstrate the willingness to solve their problem(s) collectively through a pooled procurement mechanism. This willingness includes political will, leadership, and ownership by relevant individuals or organizations.* | *(1,9,10,13,14)* |
| 1. Alignment on goals, purpose and operations of the pooled procurement mechanism (shared plan) | *The buyers need to align on goals, purpose and operations of the pooled procurement mechanism. This does not necessarily mean that all buyers need to have the same goals, purpose and operations for the mechanism. As long as they are not conflicting.* | (8,13,15–18) |
| 1. Joint need for specific products (product alignment) | *The buyers need to have a joint need for specific products. If there is no joint need, pooling around specific type of products cannot take place, and therefore buyers will lose the financial benefits resulting from economies of scale.* | *(5,19)* |
| 1. Sufficient market size to attract suppliers (market size) | *The buyers combined need to have a sufficient market size to attract suppliers for a favourable price.* | *(6,8)* |
| 1. Sufficient and stable financial capacity (financial capacity) | *The buyers combined need to have sufficient and stable financial capacity to procure through the pooled procurement mechanism.* | (8) |
| 1. Regulatory harmonization (e.g., shared quality standards, joint assessment, mutual recognition, etc.) | *Sellers are potentially attracted to pooled procurement systems because it increases their market size while reducing the need to re-register products in each country, an expensive and time-consuming proposition. If there is no shared system for approving products and allowing access to markets, then this advantage is lost in practice.* | (10,20–22) |
| 1. Trust (in other buyers and the pooled procurement organization) | *Buyers need to reach a certain level of trust in each other and the pooled procurement organization for them to share data and allocate budget. Trust levels can grow over time and can be reinforced by positive experiences with the pooled procurement mechanism.* | *(4,11,18)* |
| 1. Transparent data and information sharing | *To reduce information asymmetry, the buyers need to have a mechanism in place that allows for transparent data and information sharing on suppliers, prices and demand forecasts between each other. A lack of transparency will negatively affect trust between buyers, and will result in the pooled procurement mechanism not reaching its full potential.* | *(11,13,18)* |
| 1. No history of conflict or failed collaboration | *The level of trust between buyers might be negatively affected if the buyers share a history of conflict or a history of failed collaboration.* | *(11,23)* |
| 1. Homogeneity of buyer's characteristics related to their needs | *Buyers need to share similar characteristics (e.g., market size, demographics, financial capacity, bureaucratic structures, etc.) related to their collective needs (e.g., type of products, motivations, goals, etc.). If there is no homogeneity between buyers related to their needs, there is a greater possibility of conflicting interests within the pooled procurement mechanism, which might negatively affect the sustainability of the mechanism.* | *(3,6,24)* |
| **If buyer's mechanism, all buyers combined, nice to have:** | |  |
| 1. Shared cultural factors and values (e.g., language, traditions, etc.) | *Buyers sharing similar cultural factors and values (e.g., language, traditions, etc.) are more likely to trust and understand each other and their way of working/interacting, which will benefit the pooled procurement mechanism.* | *(4,6,7,12)* |
| 1. Existing political or structural mechanisms | *Buyers having pre-existing political or structural mechanisms in place are more likely to trust and understand each other and their way of working/interacting. These pre-existing political or structural mechanisms do not have to be limited to the area of medicine procurement. A greater level of interdependence between buyers to solve their problem(s) in other areas will stimulate buyers’ collaboration and adherence to the mechanism.* | *(12,25)* |
| 1. **Pooled procurement organization** |  |  |
| 1. Organizational and good governance structure with clear roles and responsibilities | *The pooled procurement organization needs an organizational and good governance structure with clear roles and responsibilities that buyers and suppliers trust to do business with* | (8–10,13,26) |
| 1. Clear mandate | *The pooled procurement organization needs a clear mandate that is provided by the buyers on whose behalf they procure* | *(13,27,28)* |
| 1. Standardized and transparent procedures | *The pooled procurement organization needs standardized and transparent procurement procedures to increase trust and user-friendliness* | *(1,6,11,13,28)* |
| 1. Sufficient, predictable and timely budget, either internal (through service fees) or external (through donors) to carry out pooled procurement | *The pooled procurement organization needs sufficient, predictable and timely budget to procure medicine, to attract suppliers, and to respond to unforeseen circumstances with sufficient financial buffers.* | *(8,9,23)* |
| 1. Sufficient, predictable and timely budget, either internal (through service fees) or external (through donors), to cover organizational expenses | *The pooled procurement organization needs sufficient, predictable and timely budget to cover their organizational expenses, including salaries, insurances and rental leases.* | *(8,10)* |
| 1. Predictable, timely and efficient payment mechanism | *Sellers will be more attracted to the pooled procurement mechanism if the pooled procurement organization has a predictable, timely and efficient payment mechanism, including single source payment, single currency, acceptable payment period, upfront payment.* | *(7,9,29,30)* |
| 1. Human resources (sufficient in numbers and expertise) | *The pooled procurement organization needs sufficient and expert human resources. This also means that the pooled procurement organization needs sufficient budget to provide competitive salaries to attract skilful staff.* | *(8,9)* |
| 1. Sufficient technical capacity (e.g., procurement, quality assessment, forecasting, etc.) | *The pooled procurement organization needs sufficient technical capacity to carry out tenders, to assess quality of products, to aggregate demand data, to provide capacity building for buyers, etc.* | *(8,9,27)* |
| 1. Positive reputation | *The pooled procurement organization needs to develop a positive reputation, which is based on trust from other actors in the pooled procurement mechanism. Positive reputation is necessary to attract and be entrusted with funding from buyers and funders. This positive reputation is reinforced by providing a rounded procurement service to its buyers and suppliers, including capacity building, risk sharing, market shaping, responsiveness, accountability and transparency.* | *(11,15,26,27)* |
| 1. No conflict of interest | *The staff at the pooled procurement organization should have no conflict of interest. The organization should operate independently, maximizing the benefits for all its buyers.* | *(8,28)* |
| 1. "User-friendliness" (both towards buyers and sellers) | *The pooled procurement organization should provide services to buyers and suppliers in a user-friendly manner with a reliable management information system. The benefits of procuring and supplying through the pooled procurement organization should outweigh the costs, both in terms of finances and effort.* | *(2,18)* |
| 1. **Suppliers** |  |  |
| 1. In the case of generic medicines, sufficient number of qualified suppliers | *In the case of generic medicines, an effective pooled procurement mechanism needs a sufficient number of qualified suppliers in the market. If there is no sufficient number of qualified suppliers, there will be no healthy competition among suppliers, threatening the security of supply. A potential way of overcoming the lack of a sufficient number of qualified suppliers is for the pooled procurement organization to incentive suppliers for production and supply.* | *(11)* |
| 1. Sufficient production incentives | *Incentives for suppliers to produce products for the pooled procurement organization include a consolidated and sufficient market size; warehouse(s) providing buffer stock; take-off agreements; long-term framework agreements; multiple-buyer tenders* | *(8,11,31)* |
| 1. Sufficient supply incentives | *Incentives for suppliers to supply/sell products to the pooled procurement organization include predictable timely and efficient payment mechanism; regulatory harmonization; user-friendliness; positive reputation* | (6–9,11,14,18,30) |
| 1. Sufficient number of distributors with favourable delivery terms | *An effective pooled procurement mechanism needs a sufficient number of distributors/logistics companies that are willing to deliver the product(s) for favourable delivery terms (including lead time, costs, incoterms)* | *(6)* |

# References

1. DeRoeck D, Bawazir SA, Carrasco P, Kaddar M, Brooks A, Fitzsimmons J, et al. Regional group purchasing of vaccines: review of the Pan American Health Organization EPI revolving fund and the Gulf Cooperation Council group purchasing program. Int J Health Plann Manag. 2006;21(1):23–43.

2. World Health Organization. Regional workshop on strengthening quantification and procurement of essential medicines. Report of the workshop held in New Delhi, India, 10-12 June 2014. 2014; Available from: https://apps.who.int/iris/handle/10665/206213

3. Nollet J, Beaulieu M. Should an organisation join a purchasing group? Supply Chain Manag. 2005. 1;10(1):11–7.

4. Polychronakis YE, Syntetos AA. ‘Soft’ supplier management related issues: An empirical investigation. Int J Prod Econ. 2007. 1;106(2):431–49.

5. Carrera P, Katik S, Schotanus F. Joint procurement of complex products: actual price savings, perceived nonmonetary advantages, disadvantages and impediments. J Public Procure. 2021. 1;21(2):167–82.

6. Vogler S, Haasis MA, van den Ham R, Humbert T, Garner S, Suleman F. European collaborations on medicine and vaccine procurement. Bull World Health Organ. 2021. 1;99(10):715–21.

7. Onyango C, Aboagye-Nyame F. Readiness for Regional Pooled Procurement of HIV/AIDS-Related Drugs and Commodities in Sub-Saharan Africa: An Assessment of 11 Member Countries of the Commonwealth Regional Health Community Secretariat, 2002. Management Sciences for Health; 2003.

8. Parmaksiz K, Pisani E, Bal R, Kok MO. A systematic review of pooled procurement of medicines and vaccines: identifying elements of success. Global Health. 2022. 11;18(1):59.

9. Domfeh KA. Pooled procurement program in the quality improvement of medicines of the National Catholic Health Service in Ghana: using the Donabedian model. J Pharm Health Serv Res. 2021. 1;12(2):133–41.

10. Hany Abdallah. West Africa Reproductive Health Commodity Security: Review of Pooled Procurement. John Snow, Inc./DELIVER; 2005. Available from: https://www.rhsupplies.org/uploads/tx_rhscpublications/DELIVER_Pooled%20Procurement%20Review_West%20Africa_2007.pdf

11. Pazirandeh A, Herlin H. Unfruitful cooperative purchasing: A case of humanitarian purchasing power. J Humanit Logist Supply Chain Manag. 2014. 1;4(1):24–42.

12. Baute S, de Ruijter A. EU health solidarity in times of crisis: explaining public preferences towards EU risk pooling for medicines. J Eur Public Policy. 2022. 3;29(8):1183–205.

13. World Health Organization Regional Office for Europe. Cross-country collaborations to improve access to medicines and vaccines in the WHO European Region. Copenhagen, Denmark: World Health Organization. Regional Office for Europe; 2020. Available from: https://apps.who.int/iris/handle/10665/332933

14. Huff-Rousselle M. The logical underpinnings and benefits of pooled pharmaceutical procurement: a pragmatic role for our public institutions? Soc Sci Med. 2012;75(9):1572–80.

15. Dornan M, Newton Cain T. Regional Service Delivery among Pacific Island Countries: An Assessment: Pacific regionalism. Asia Pac Policy Stud. 2014;1(3):541–60.

16. Schotanus F, Telgen J, Boer L de. Critical success factors for managing purchasing groups. J Purch Supply Manag. 2010. 1;16(1):51–60.

17. Cherla A, Howard N, Mossialos E. The ‘Netflix plus model’: can subscription financing improve access to medicines in low- and middle-income countries? Health Econ Policy Law. 2020. 3;1–11.

18. Gobbi C, Hsuan J. Collaborative purchasing of complex technologies in healthcare: Implications for alignment strategies. Int J Oper Prod Manag. 2015;35(3):430–55.

19. Schotanus F. Cooperative purchasing within the United Nations. In Archamps, France; 2005. Available from: https://ris.utwente.nl/ws/portalfiles/portal/5524139/Schotanus_-_Cooperative_Purchasing_UN.pdf

20. Management Science for Health. MDS-3: Managing Access to Medicines and Health Technologies (Third Edition). Arlington, VA: Management Science for Health; 2012. Available from: http://apps.who.int/medicinedocs/documents/s19577en/s19577en.pdf

21. UNECA. The AfCFTA: Opportunities for pooled procurement of essential drugs and products and local pharmaceutical production for the continent. 2019. Available from: https://www.uneca.org/sites/default/files/afcfta-pharma-initiative/afcfta-pharma-event-2019/pharma_high_level_meeting_report_final.pdf

22. Alix Beith, Nadia Olson, Wendy Abramson. Regulatory Harmonization in Central America: How Harmonization Can Impact Regional Contraceptive Procurement. 2007. Available from: https://www.rhsupplies.org/uploads/tx_rhscpublications/DELIVER_Regulatory%20Harmonsition%20&%20Contraceptive%20Procurement_Central%20America_2007.pdf

23. Vaillancourt A. Procurement consolidation in humanitarian supply chains: A case study. Int J Procure Manag. 2017;10(2):178–93.

24. Chalkidou K, Claxton K, Silverman R, Yadav P. Value-based tiered pricing for universal health coverage: An idea worth revisiting. Gates Open Res. 2020;4.

25. Azzopardi-Muscat N, Schroder-Back P, Brand H. The European Union Joint Procurement Agreement for cross-border health threats: what is the potential for this new mechanism of health system collaboration? Health Econ Policy Law. 2017 Jan;12(1):43–59.

26. Kuwawenaruwa A, Tediosi F, Obrist B, Metta E, Chiluda F, Wiedenmayer K, et al. The role of accountability in the performance of Jazia prime vendor system in Tanzania. J Pharm Policy Pract. 2020 Jun 8;13(1):25.

27. Kumaresan J, Smith I, Arnold V, Evans P. The Global TB Drug Facility: innovative global procurement. Int J Tuberc Lung Dis. 2004;8(1):130–8.

28. MAPS Initiative. Methodology for Assessing Procurement Systems (MAPS). 2018. Available from: https://www.mapsinitiative.org/methodology/MAPS-methodology-for-assessing-procurement-systems.pdf

29. Huff-Rousselle M, Burnett F. Cost containment through pharmaceutical procurement: A Caribbean case study. I nt J Health Plann Manag. 1996;11(2):135–57.

30. Neumann I, Schünemann HJ, Bero L, Cooke G, Magrini N, Moja L. Global access to affordable direct oral anticoagulants. Bull World Health Organ. 2021. 1;99(9):653–60.

31. USAID Center for Accelerating Innovation and Impact. Healthy Markets for Global Health: A Market Shaping Primer. 2014. Available from: https://www.usaid.gov/sites/default/files/documents/1864/healthymarkets_primer.pdf
